# Supplementary material for: Creation of a Prognostic Model Using Cuproptosis-Associated Long Noncoding RNAs in Hepatocellular Carcinoma
Source: Int J Mol Sci. 2023 Jun 10;24(12):9987. doi: 10.3390/ijms24129987 (PMC10298112; doi:10.3390/ijms24129987)
Supplement: Supplementary file 1 [file ijms-24-09987-s001.zip › ijms-2342103-supplementary.pdf]

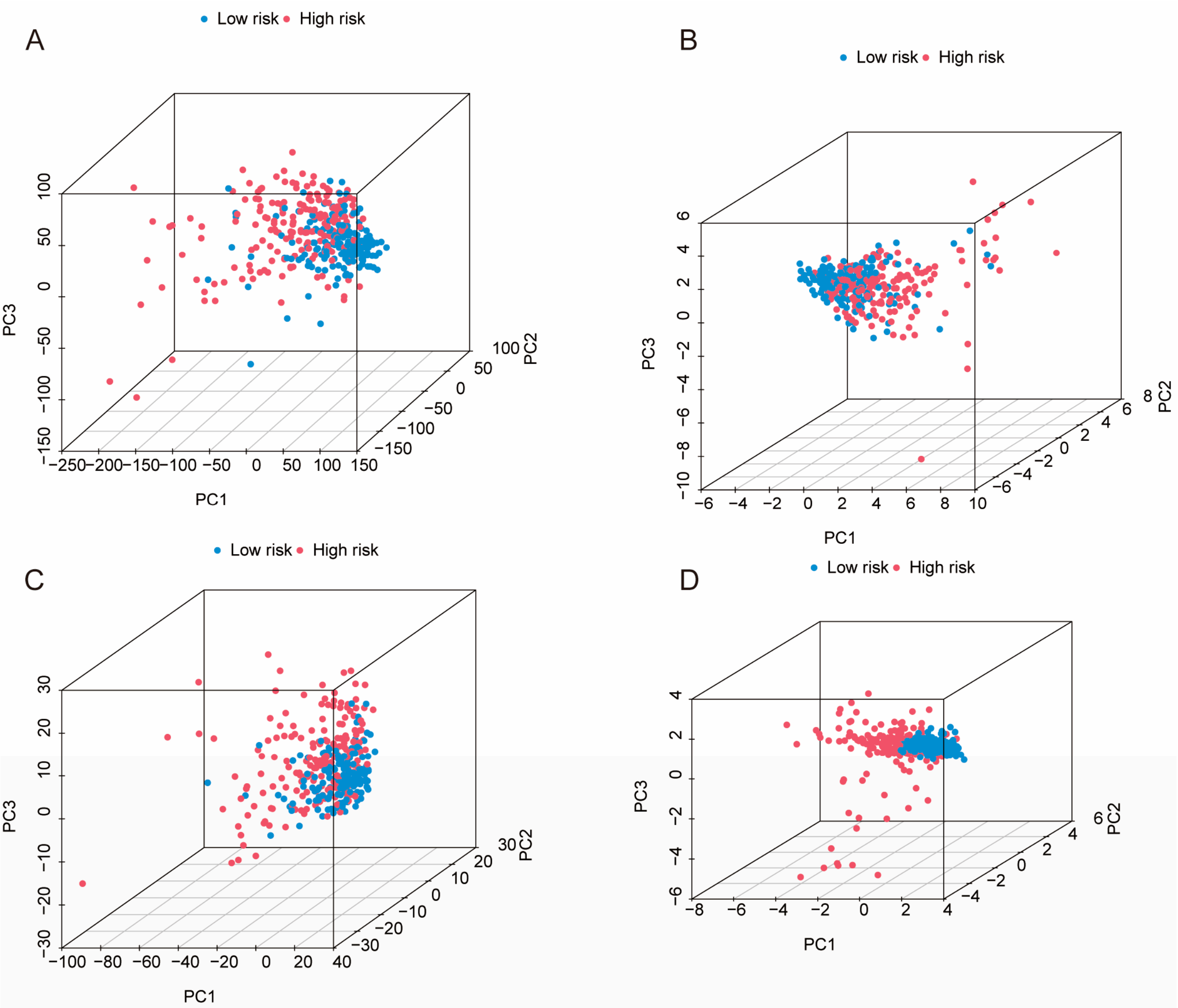


**Figure S1.** PCA of high-risk and low-risk groups. **(A)** Entire gene expression profiles. **(B)** Nineteen cuproptosis-related genes. **(C)** Cuproptosis-related lncRNAs. **(D)** Risk model based on the 7-cuproptosis-related lncRNAs.

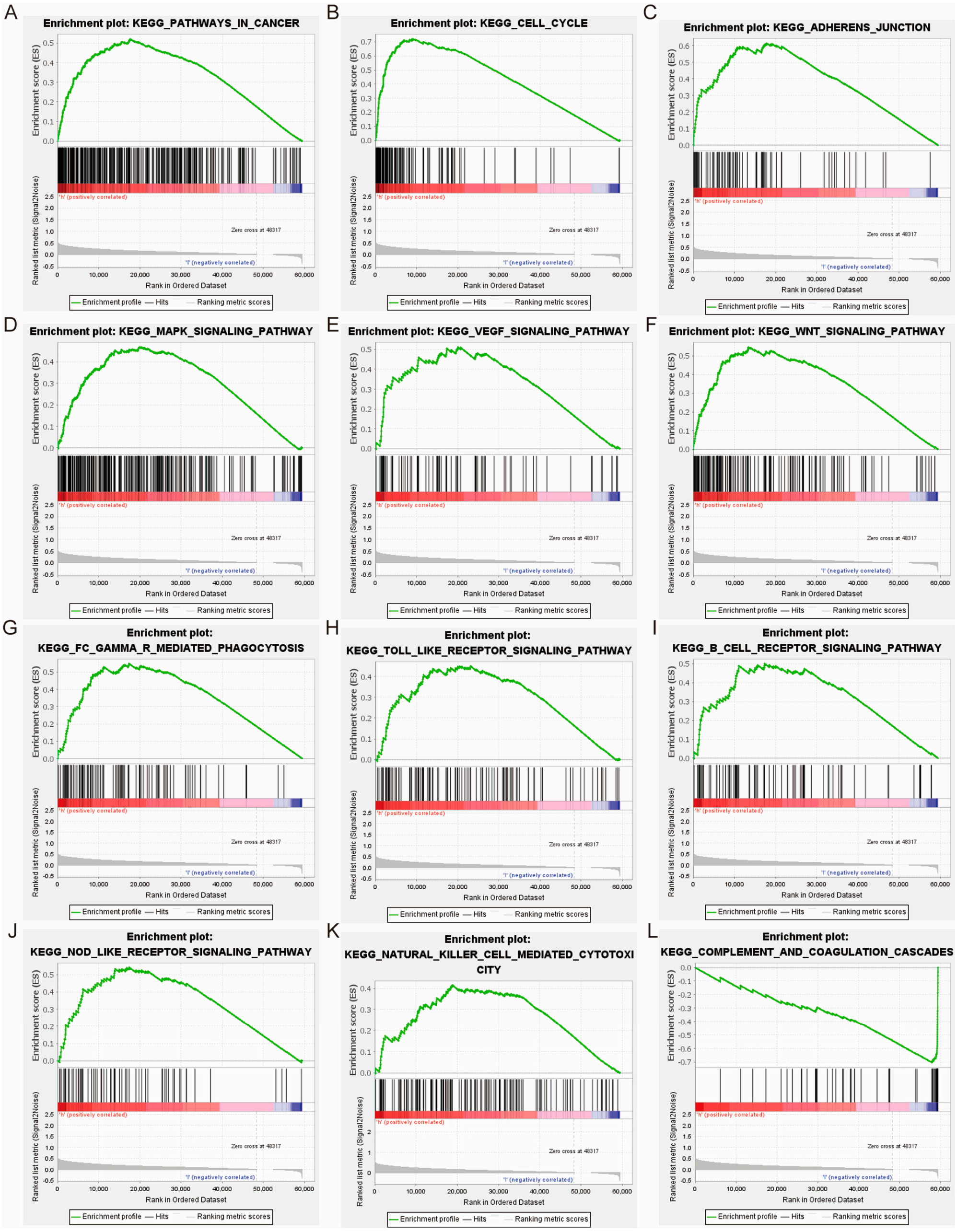


**Figure S2.** GSEA enrichment analysis. **(A-F)** High-risk group was associated with the occurrence and development of LIHC. **(G-L)** Risk model associated with immune pathways.

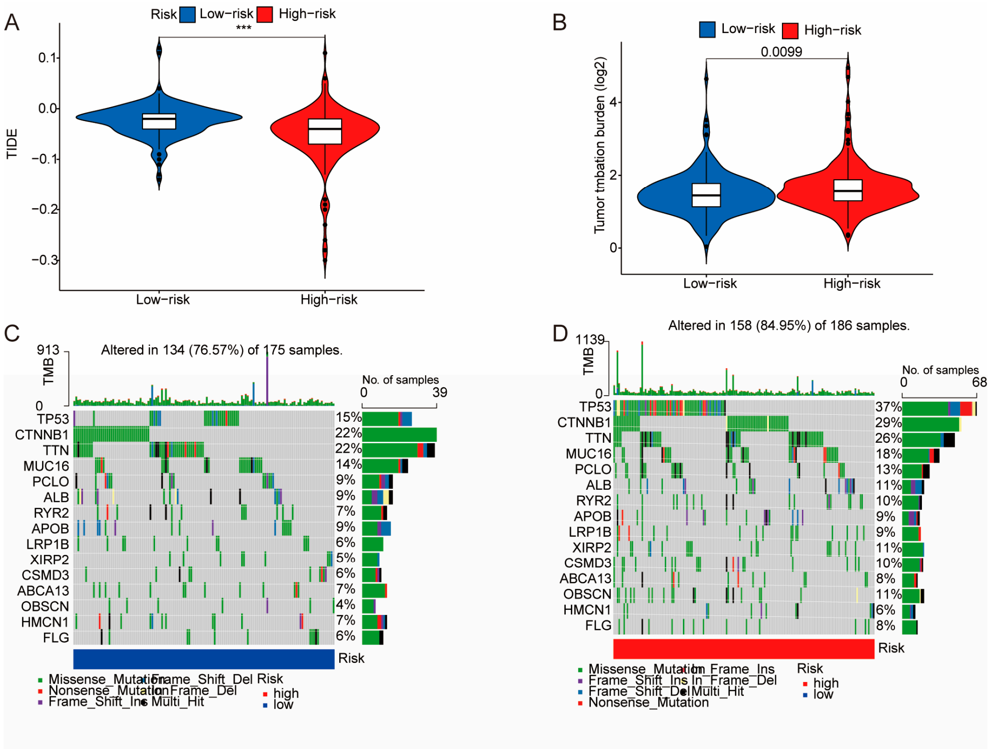


**Figure S3.** Assessing the response of the risk model to tumor immunotherapy. **(A)** The high-risk group had significantly lower TIDE scores than the low-risk group. **(B)** The high-risk group had significantly higher TMB than the low-risk group. **(C-D)** The waterfall plot presented the genes with the highest mutation frequency in the high-risk and low-risk groups, respectively.

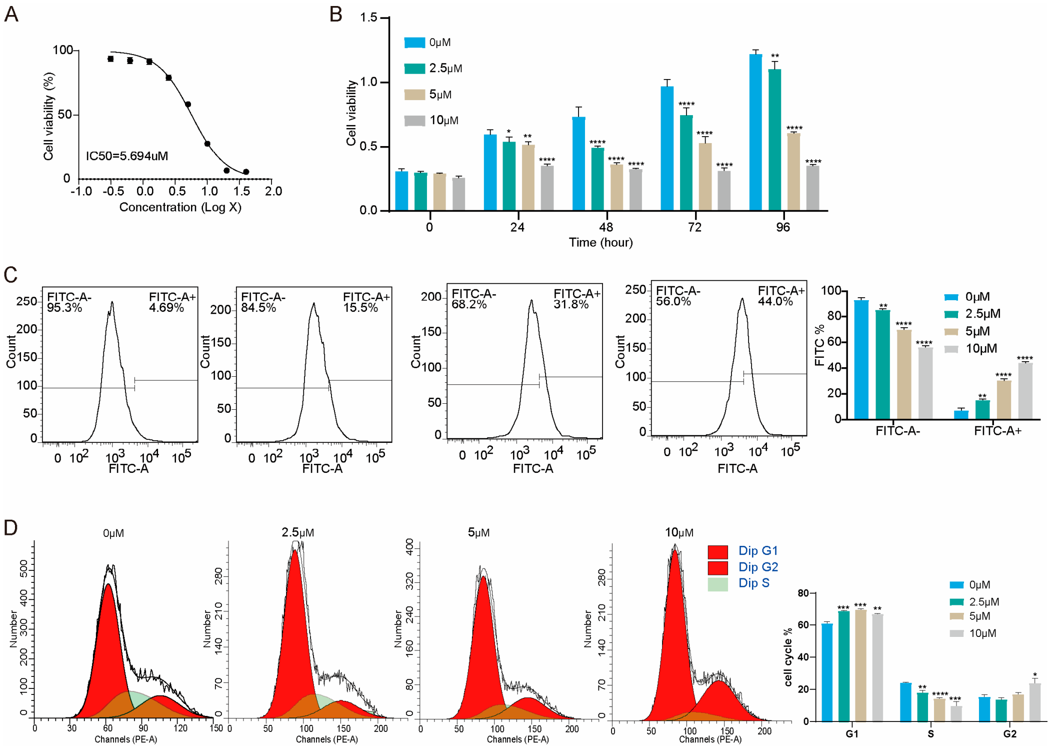


**Figure S4**. Sorafenib inhibited the proliferation and cycle of huh7 cells. **(A)** The $IC_{50}$ value of Sorafenib in the Huh7 cells. **(B)** Different concentrations of Sorafenib (0 μM, 2.5 μM, 5 μM, 10 μM) inhibited the

proliferation of Huh7 cells through CCK8 assay, and the data were calculated at 0, 24, 48 and 72 hours. **(C)** The CFSE assay showed that the different concentrations of Sorafenib (0 μM, 2.5 μM, 5 μM, 10 μM) inhibited the proliferation of Huh7 cells. **(D)** Different concentrations of Sorafenib (0 μM, 2.5 μM, 5 μM, 10 μM) blocked cell cycle to G1 stage. All * $p_{value} < 0.05$, ** $p_{value} < 0.01$, *** $p_{value} < 0.001$, **** $p_{value} < 0.0001$.

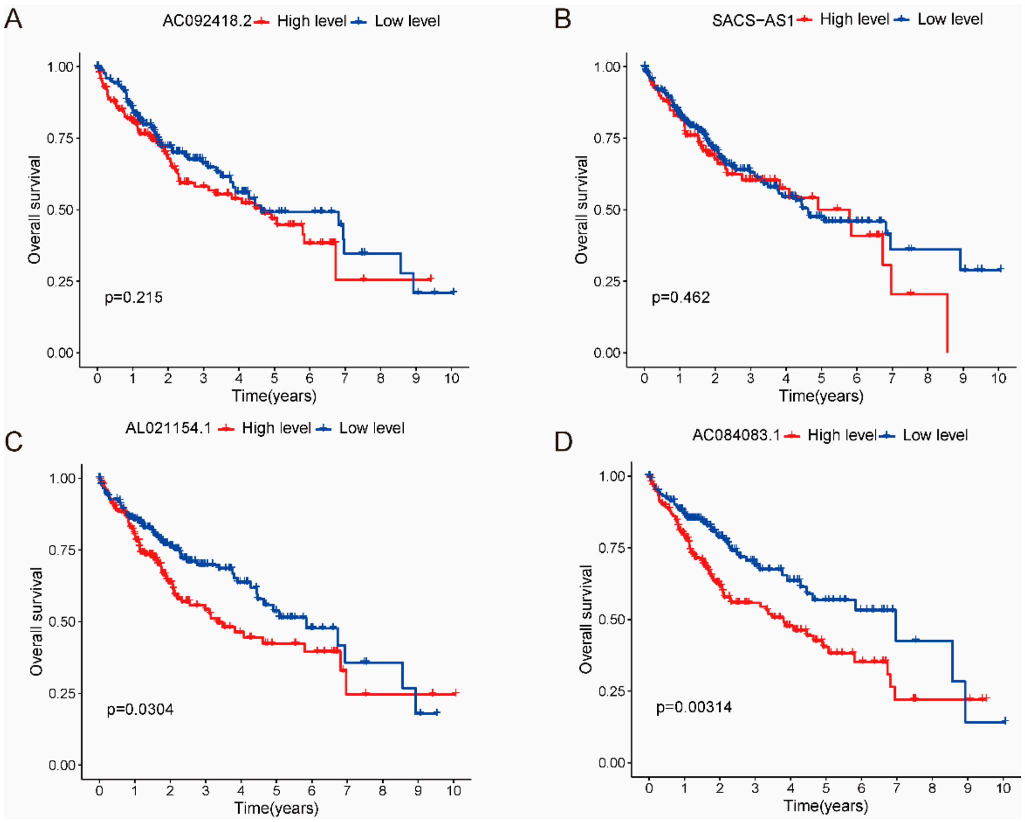


**Figure S5**. The Kaplan-Meier survival curves. (**A-D**) The survival rate of lncRNAs (AC092418.2, SACS-AS1, AL021154.1 and AC084083.1) in HCC patients.

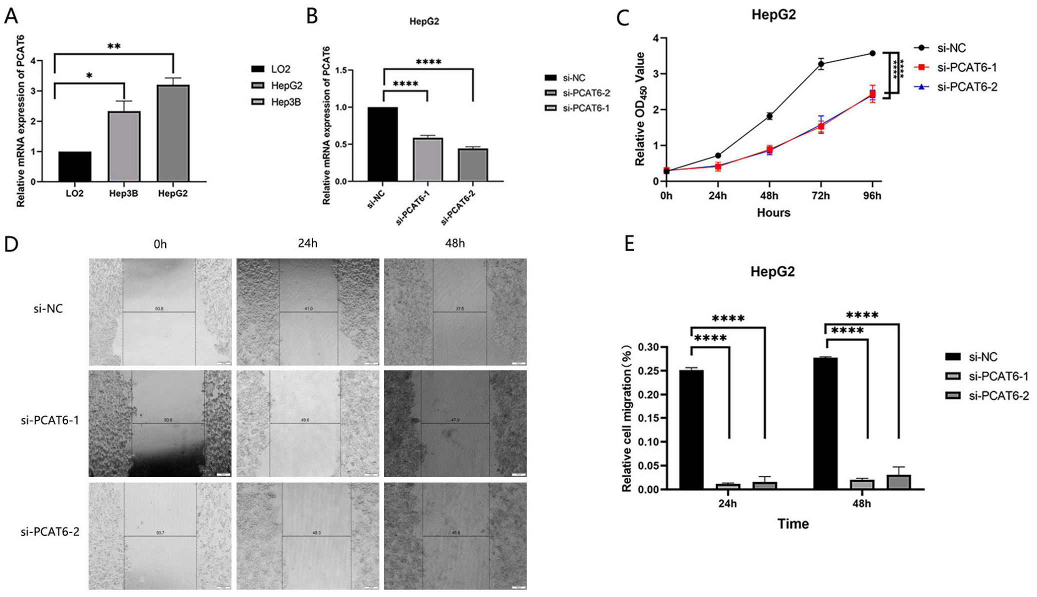

**Figure S6.** Inhibition of PCAT6 restrained proliferation and migration capacities of HCC cells. **(A)** Expression levels of PCAT6 were detected in HCC cell lines (HepG2 and Hep3B) and normal hepatic epithelium cell lines (LO2). **(B)** RT-qPCR analysis revealed the efficiency of PCAT6 knocking down in HepG2 cell lines. **(C)** CCK-8 cell proliferation assay after PCAT6 knockdown HepG2 cell lines. **(D)** The cell scratch test results showed that the knocking-down of PCAT6 inhibited the cellular migration of HepG2. **(E)** The data were calculated as the relative cell migration (%) at 0 hour, 24 hours, and 48 hours. All * $p_{\text{value}} < 0.05$, ** $p_{\text{value}} < 0.01$, **** $p_{\text{value}} < 0.0001$.

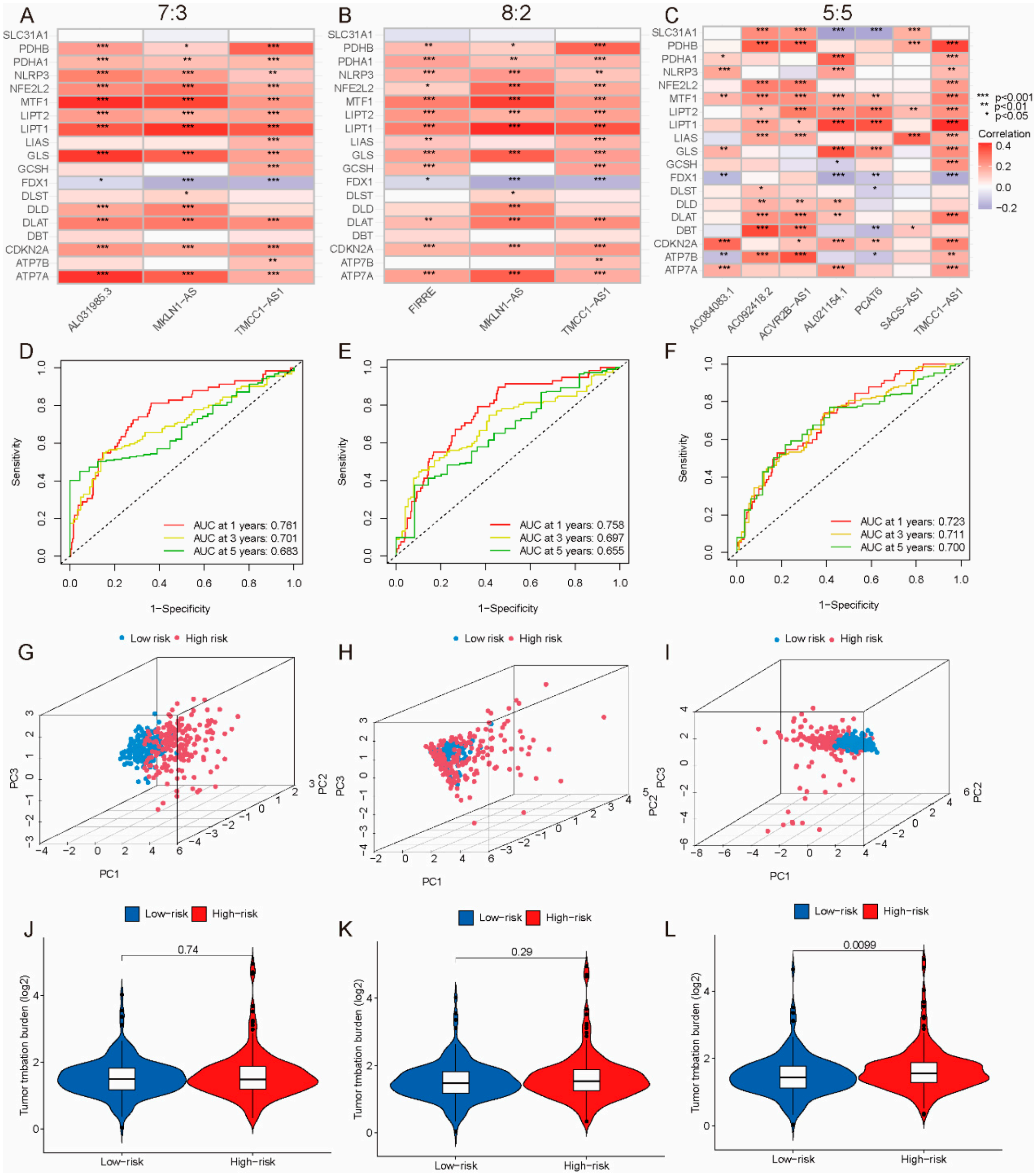


**Figure S7**. Comparison of different risk models. **(A)** Heatmap of the association between 19 cuproptosis-related genes and three lncRNAs with the ratio of 7:3. **(B)** Heatmap of the association between 19 cuproptosis-related genes and three lncRNAs with the ratio of 8:2. **(C)** Heatmap of the association between 19 cuproptosis-related genes and three lncRNAs with the ratio of 5:5. **(D)** The ROC curve plots. The accuracy of risk models for patients at 1-, 3- and 5 years with the ratio of

7:3. **(E)** The ROC curve plots. The accuracy of risk models for patients at 1-, 3- and 5 years with the ratio of 8:2. **(F)** The ROC curve plots. The accuracy of risk models for patients at 1-, 3- and 5 years with the ratio of 5:5. **(G)** PCA of high-risk and low-risk groups with the ratio of 7:3. **(H)** PCA of high-risk and low-risk groups with the ratio of 8:2. **(I)** PCA of high-risk and low-risk groups with the ratio of 5:5. **(J)** The high-risk group and low-risk group had no difference TMB with the ratio of 7:3. **(K)** The high-risk group and low-risk group had no difference TMB with the ratio of 8:2. **(L)** The high-risk group had significantly higher TMB than the low-risk group with the ratio of 5:5.

**Table S1.** Top 100 GSEA enrichment results for the high-risk group.

| GSEA enrichment | ES | NES | P-value |
|---|---|---|---|
| KEGG_RNA_ DEGRADATION | 0.76 | 2.03 | 0 |
| KEGG_OOCYTE_ MEIOSIS | 0.65 | 2.02 | 0 |
| KEGG_BASAL_ TRANSCRIPTION_ FACTORS | 0.75 | 1.98 | 0 |
| KEGG_ SPLICEOSOME | 0.79 | 1.98 | 0 |
| KEGG_CELL_ CYCLE | 0.72 | 1.96 | 0 |
| KEGG_ SELENOAMINO_ ACID_METABOLISM | 0.69 | 1.94 | 0 |
| KEGG_NUCLEOTIDE_ EXCISION_REPAIR | 0.75 | 1.93 | 0 |
| KEGG_UBIQUITIN_ MEDIATED_ PROTEOLYSIS | 0.69 | 1.92 | 0 |
| KEGG_AMINOACYL_ TRNA_BIOSYNTHESIS | 0.74 | 1.91 | 0.002 |
| KEGG_PYRIMIDINE_ METABOLISM | 0.61 | 1.9 | 0 |
| KEGG_ REGULATION_ OF_AUTOPHAGY | 0.63 | 1.89 | 0 |
| KEGG_PURINE_ METABOLISM | 0.57 | 1.89 | 0 |
| KEGG_BASE_ EXCISION_REPAIR | 0.72 | 1.89 | 0 |
| KEGG_ HOMOLOGOUS_ RECOMBINATION | 0.78 | 1.88 | 0 |
| KEGG_PROGESTERONE_ MEDIATED_OOCYTE_ MATURATION | 0.62 | 1.87 | 0.002 |
| KEGG_N_ GLYCAN_BIOSYNTHESIS | 0.68 | 1.84 | 0.002 |
| KEGG_ENDOCYTOSIS | 0.59 | 1.83 | 0 |

| | | | |
|---|---|---|---|
| KEGG_INSULIN_ SIGNALING_PATHWAY | 0.54 | 1.77 | 0.002 |
| KEGG_CYSTEINE_ AND_METHIONINE_ METABOLISM | 0.57 | 1.76 | 0.006 |
| KEGG_ NEUROTROPHIN_ SIGNALING_PATHWAY | 0.6 | 1.76 | 0.004 |
| KEGG_DNA_REPLICATION | 0.77 | 1.76 | 0.008 |
| KEGG_GLYCOSYL_ PHOSPHATIDYLINOSITOL_ GPI_ANCHOR_BIOSYNTHESIS | 0.69 | 1.73 | 0 |
| KEGG_ADHERENS_JUNCTION | 0.61 | 1.73 | 0.002 |
| KEGG_WNT_SIGNALING_ PATHWAY | 0.55 | 1.73 | 0.002 |
| KEGG_MTOR_SIGNALING_ PATHWAY | 0.6 | 1.73 | 0.006 |
| KEGG_VASOPRESSIN_ REGULATED_WATER_ REABSORPTION | 0.64 | 1.72 | 0.002 |
| KEGG_CHRONIC_ MYELOID_LEUKEMIA | 0.61 | 1.71 | 0.006 |
| KEGG_P53_SIGNALING_ PATHWAY | 0.56 | 1.71 | 0.006 |
| KEGG_LYSINE_ DEGRADATION | 0.61 | 1.71 | 0.014 |
| KEGG_PATHWAYS_ IN_CANCER | 0.52 | 1.7 | 0.006 |
| KEGG_MISMATCH_ REPAIR | 0.75 | 1.69 | 0.006 |
| KEGG_SNARE_ INTERACTIONS_IN_ VESICULAR_TRANSPORT | 0.62 | 1.68 | 0.008 |
| KEGG_NOTCH_ SIGNALING_PATHWAY | 0.61 | 1.68 | 0.006 |
| KEGG_PATHOGENIC_ ESCHERICHIA_COLI_ INFECTION | 0.59 | 1.68 | 0.02 |
| KEGG_RNA_ POLYMERASE | 0.66 | 1.67 | 0.008 |
| KEGG_THYROID_ CANCER | 0.61 | 1.66 | 0.01 |
| KEGG_SPHINGOLIPID_ METABOLISM | 0.58 | 1.66 | 0.006 |
| KEGG_ERBB_ SIGNALING_PATHWAY | 0.56 | 1.65 | 0.01 |
| KEGG_COLORECTAL_ CANCER | 0.59 | 1.65 | 0.016 |
| KEGG_ MELANOGENESIS | 0.5 | 1.64 | 0.01 |
| KEGG_ | 0.58 | 1.63 | 0.026 |

| | | | |
|---|---|---|---|
| ENDOMETRIAL_CANCER | | | |
| KEGG_RIG_I_LIKE_ RECEPTOR_SIGNALING_ PATHWAY | 0.54 | 1.63 | 0.016 |
| KEGG_INOSITOL_ PHOSPHATE_ METABOLISM | 0.59 | 1.62 | 0.02 |
| KEGG_LONG_TERM_ POTENTIATION | 0.53 | 1.62 | 0.018 |
| KEGG_NON_ SMALL_CELL_ LUNG_CANCER | 0.56 | 1.62 | 0.02 |
| KEGG_AMINO_SUGAR_ AND_NUCLEOTIDE_ SUGAR_METABOLISM | 0.54 | 1.62 | 0.014 |
| KEGG_ GLYCEROPHOSPHOLIPID_ METABOLISM | 0.48 | 1.61 | 0.004 |
| KEGG_ GLYCOSPHINGOLIPID_ BIOSYNTHESIS_ GANGLIO_SERIES | 0.64 | 1.61 | 0.019 |
| KEGG_PANCREATIC_ CANCER | 0.58 | 1.61 | 0.035 |
| KEGG_VEGF_ SIGNALING_PATHWAY | 0.51 | 1.6 | 0.018 |
| KEGG_SMALL_CELL_ LUNG_CANCER | 0.55 | 1.59 | 0.029 |
| KEGG_PROSTATE_ CANCER | 0.53 | 1.59 | 0.035 |
| KEGG_ONE_CARBON_ POOL_BY_FOLATE | 0.58 | 1.59 | 0.03 |
| KEGG_GLIOMA | 0.53 | 1.58 | 0.025 |
| KEGG_ACUTE_ MYELOID_LEUKEMIA | 0.56 | 1.58 | 0.034 |
| KEGG_OTHER_ GLYCAN_DEGRADATION | 0.63 | 1.58 | 0.032 |
| KEGG_TIGHT_JUNCTION | 0.48 | 1.57 | 0.028 |
| KEGG_GNRH_ SIGNALING_PATHWAY | 0.5 | 1.57 | 0.03 |
| KEGG_RENAL_ CELL_CARCINOMA | 0.56 | 1.57 | 0.049 |
| KEGG_AXON_ GUIDANCE | 0.51 | 1.56 | 0.031 |
| KEGG_FC_GAMMA_ R_MEDIATED_ PHAGOCYTOSIS | 0.55 | 1.56 | 0.049 |
| KEGG_LONG_ TERM_DEPRESSION | 0.49 | 1.56 | 0.027 |
| KEGG_LYSOSOME | 0.52 | 1.55 | 0.04 |
| KEGG_APOPTOSIS | 0.53 | 1.55 | 0.032 |

| | | | |
|---|---|---|---|
| KEGG_ PHOSPHATIDYLINOSITOL_ SIGNALING_SYSTEM | 0.55 | 1.55 | 0.039 |
| KEGG_TGF_BETA_ SIGNALING_PATHWAY | 0.52 | 1.55 | 0.037 |
| KEGG_EPITHELIAL_ CELL_SIGNALING_IN_ HELICOBACTER_ PYLORI_INFECTION | 0.53 | 1.54 | 0.034 |
| KEGG_FC_EPSILON_ RI_SIGNALING_PATHWAY | 0.5 | 1.54 | 0.028 |
| KEGG_MAPK_ SIGNALING_PATHWAY | 0.47 | 1.54 | 0.032 |
| KEGG_BLADDER_CANCER | 0.54 | 1.53 | 0.052 |
| KEGG_VIBRIO_ CHOLERAE_INFECTION | 0.52 | 1.53 | 0.025 |
| KEGG_HUNTINGTONS_ DISEASE | 0.46 | 1.51 | 0.055 |
| KEGG_AMYOTROPHIC_ LATERAL_SCLEROSIS_ALS | 0.47 | 1.5 | 0.025 |
| KEGG_NOD_LIKE_ RECEPTOR_SIGNALING_ PATHWAY | 0.54 | 1.5 | 0.06 |
| KEGG_PROTEASOME | 0.59 | 1.49 | 0.085 |
| KEGG_REGULATION_ OF_ACTIN_CYTOSKELETON | 0.47 | 1.49 | 0.063 |
| KEGG_FRUCTOSE_AND_ MANNOSE_METABOLISM | 0.48 | 1.48 | 0.039 |
| KEGG_BASAL_CELL_ CARCINOMA | 0.48 | 1.46 | 0.05 |
| KEGG_PENTOSE_ PHOSPHATE_PATHWAY | 0.5 | 1.45 | 0.063 |
| KEGG_GAP_JUNCTION | 0.48 | 1.45 | 0.083 |
| KEGG_NICOTINATE_ AND_NICOTINAMIDE_ METABOLISM | 0.49 | 1.44 | 0.07 |
| KEGG_T_CELL_ RECEPTOR_ SIGNALING_PATHWAY | 0.51 | 1.44 | 0.105 |
| KEGG_PROTEIN_ EXPORT | 0.57 | 1.44 | 0.1 |
| KEGG_CYTOSOLIC_ DNA_SENSING_PATHWAY | 0.48 | 1.43 | 0.096 |
| KEGG_GLYCEROLIPID_ METABOLISM | 0.42 | 1.42 | 0.053 |
| KEGG_ADIPOCYTOKINE_ SIGNALING_PATHWAY | 0.43 | 1.42 | 0.063 |
| KEGG_ GLYCOSAMINOGLYCAN_ DEGRADATION | 0.53 | 1.41 | 0.095 |
| KEGG_TYPE_II_ | 0.45 | 1.4 | 0.094 |

| | | | |
|---|---|---|---|
| DIABETES_MELLITUS | | | |
| KEGG_LEUKOCYTE_ TRANSENDOTHELIAL_ MIGRATION | 0.45 | 1.4 | 0.129 |
| KEGG_ GLYCOSAMINOGLYCAN_ BIOSYNTHESIS_ HEPARAN_SULFATE | 0.51 | 1.4 | 0.101 |
| KEGG_B_CELL_ RECEPTOR_ SIGNALING_PATHWAY | 0.5 | 1.4 | 0.121 |
| KEGG_DORSO_ VENTRAL_AXIS_ FORMATION | 0.53 | 1.39 | 0.123 |
| KEGG_ETHER_ LIPID_METABOLISM | 0.44 | 1.38 | 0.088 |
| KEGG_HEDGEHOG_ SIGNALING_PATHWAY | 0.45 | 1.38 | 0.085 |
| KEGG_GLUTATHIONE_ METABOLISM | 0.42 | 1.37 | 0.102 |
| KEGG_JAK_STAT_ SIGNALING_PATHWAY | 0.43 | 1.37 | 0.106 |
| KEGG_TOLL_LIKE_ RECEPTOR_ SIGNALING_PATHWAY | 0.45 | 1.37 | 0.139 |
| KEGG_ ARRHYTHMOGENIC_ RIGHT_VENTRICULAR_ CARDIOMYOPATHY_ ARVC | 0.46 | 1.37 | 0.121 |
| KEGG_MELANOMA | 0.43 | 1.37 | 0.118 |
| KEGG_ GLYCOSAMINOGLYCAN_ BIOSYNTHESIS_KERATAN_ SULFATE | 0.56 | 1.34 | 0.129 |
| KEGG_FOCAL_ ADHESION | 0.43 | 1.33 | 0.178 |

**Table S2**. 119 anticancer drugs.

| Drug name | Drug name | Drug name | Drug name |
|---|---|---|---|
| 5-Fluorouracil | SN-38 | Lapatinib | EKB-569 |
| ZM-447439 | SGC0946 | KIN001-270 | EHT 1864 |
| Zibotentan | Salubrinal | KIN001-266 | Doxorubicin |
| YM201636 | Ruxolitinib | KIN001-260 | DMOG |
| Y-39983 | Roscovitine | KIN001-244 | CP724714 |
| XMD15-27 | RO-3306 | KIN001-236 | CP466722 |
| XMD11-85h | Rapamycin | KIN001-102 | CI-1040 |
| XL-184 | QL-XII-61 | KIN001-055 | CGP-60474 |
| XAV939 | Pyrimethamine | JW-7-52-1 | CGP-082996 |

| | | | |
|---|---|---|---|
| WZ-1-84 | PIK-93 | JW-7-24-1 | CEP-701 |
| VX-702 | PHA-793887 | JNK-9L | CCT007093 |
| VX-680 | PHA-665752 | Ispinesib Mesylate | BX-912 |
| Vinorelbine | PF-562271 | Imatinib | Bortezomib |
| UNC1215 | PD-173074 | I-BET-762 | BMS-509744 |
| Tubastatin A | PD-0332991 | HG-6-64-1 | BMS345541 |
| Trametinib | PD-0325901 | HG-5-88-01 | BIX02189 |
| TPCA-1 | Parthenolide | GW843682X | BI-2536 |
| TL-1-85 | Paclitaxel | GW-2580 | BHG712 |
| Tivozanib | PAC-1 | GSK690693 | Bexarotene |
| Tipifarnib | OSI-930 | GSK2126458 | BAY 61-3606 |
| THZ-2-49 | Obatoclax Mesylate | GSK1904529A | ATRA |
| Thapsigargin | NPK76-II-72-1 | GSK1070916 | AT-7519 |
| TG101348 | NG-25 | Genentech Cpd 10 | AS601245 |
| Talazoparib | MK-2206 | Gemcitabine | AP-24534 |
| TAK-715 | Midostaurin | GDC0449 | AKT inhibitor VIII |
| T0901317 | MG-132 | FR-180204 | AG-014699 |
| Sunitinib | Masitinib | Foretinib | AC220 |
| S-Trityl-L-cysteine | LY317615 | FMK | A-443654 |
| STF-62247 | LFM-A13 | Erlotinib | 681640 |
| Sorafenib | LAQ824 | Epothilone B | |
